# Combining sequence and network information to enhance protein–protein interaction prediction

**DOI:** 10.1186/s12859-020-03896-6

**Published:** 2020-12-16

**Authors:** Leilei Liu, Xianglei Zhu, Yi Ma, Haiyin Piao, Yaodong Yang, Xiaotian Hao, Yue Fu, Li Wang, Jiajie Peng

**Affiliations:** 1 College of Intelligence and Computing, Tianjin University, No.135 Yaguan Road, Tianjin, 300350 China; 2 Automotive Data Center, CATARC, No.69 Xianfeng Road, Tianjin, 300300 China; 3 School of Electronics and Information, Northwestern Polytechnical University, No.127 West Youyi Road, Xi’an, 710072 China; 4 School of Computer Science, Northwestern Polytechnical University, No.127 West Youyi Road, Xi’an, 710072 China

**Keywords:** Protein–protein interactions, Amino acid sequence, Graph convolutional networks

## Abstract

**Background:**

Protein–protein interactions (PPIs) are of great importance in cellular systems of organisms, since they are the basis of cellular structure and function and many essential cellular processes are related to that. Most proteins perform their functions by interacting with other proteins, so predicting PPIs accurately is crucial for understanding cell physiology.

**Results:**

Recently, graph convolutional networks (GCNs) have been proposed to capture the graph structure information and generate representations for nodes in the graph. In our paper, we use GCNs to learn the position information of proteins in the PPIs networks graph, which can reflect the properties of proteins to some extent. Combining amino acid sequence information and position information makes a stronger representation for protein, which improves the accuracy of PPIs prediction.

**Conclusion:**

In previous research methods, most of them only used protein amino acid sequence as input information to make predictions, without considering the structural information of PPIs networks graph. We first time combine amino acid sequence information and position information to make representations for proteins. The experimental results indicate that our method has strong competitiveness compared with several sequence-based methods.

## Background

PPIs play an important role in cellular systems of organisms, most proteins perform their functions by interacting with other proteins, so information about the PPIs can help us better understand the function of proteins [1]. Many basic cellular processes involve PPIs, for example metabolic cycles, DNA transcription and replication, and signaling cascades [2]. Disfunction in the PPIs will affect people’s health and cause diseases, research shows that many diseases are the result of abnormal PPIs involving endogenous proteins, proteins from pathogens or both [3]. Accurately predicting protein interactions is very important for us to study the properties of cellular systems, improve the understanding of disease and provide a basis for the development of novel therapeutic approaches [4].

In recent years, high-throughput biological techniques and large-scale experimental approaches for PPIs identification have achieved tremendous development, lots of PPIs data from different organisms has been discovered by researchers [2]. And *Yeast* predominantly provides PPIs data so far. But the coverage of PPIs data is still very low and there is lots of noisy data in the PPIs dataset, since experimental methods inevitably produce false-positive results [5]. According to previous researches, 50% of the *Yeast* PPIs map and only 10% of the *Human* PPIs networks have been characterised [4]. Moreover, biological techniques and large-scale experiments are often expensive, time-consuming and labor-intensive [6, 7]. Calculation-based methods can solve the problem to a certain extent, and provide reference and guidance for the biological experiment design, which are helpful for laboratory validation.

These computational methods are mainly composed of two phases, representation phase and prediction phase. In representation phase, the methods generate a vectorized representation for each protein using its attribute information. And in the prediction phase, they use traditional machine learning techniques or deep learning to make predictions based on the representation generated in the previous phase. Many characteristic properties of proteins can be used to generate representation, including protein structure information, protein domains, gene neighborhood, phylogenetic profiles, gene expression and literature mining knowledge [8]. In bioinformatics fields, STRINGDB is the most commonly used database, which collects a lot of PPIs data from different species and provides online querying and API for users to retrieve data. For PPIs annotations, STRINGDB computes a combined score by combining the probabilities from the different evidence channels, including fusion evidence, neighborhood evidence, cooccurrence evidence, experimental evidence, textmining evidence, database evidence and coexpression evidence [9]. However, the design of above representation methods requires strong domain knowledge, and some information is difficult to obtain, which limits the practicality of the method to a certain extent [8]. Recently, protein amino acid sequence data has a rapid growth. Compared with the limited number of protein structures, it is undeniable that the number of protein sequences is much larger. Computational methods that make predictions only based on amino acid sequence are arousing great interest of researchers. The experimental results in previous works show that only using protein sequence information can also achieve high prediction accuracy [2, 5, 7, 8, 10–13].

These sequence-based methods have achieved certain results, but the method of generating vectorized representation for proteins based on protein sequence information is complicated. And some methods need extra statistical information, computation complexity and time complexity are high. Moreover, they did not use the structural information of the PPIs networks graph. PPIs data can be represented in the form of graph, where nodes represent proteins and edges represent protein interactions. So the position information of protein in the graph, which can also be said to be the relationship between proteins, can reflect the properties of proteins to some extent, which is an important supplement to protein sequence information. Combining amino acid sequence information and position information can help to make a more accurate prediction. In this paper, we first time use GCNs to capture the protein’s position information in the PPIs networks graph and combine the amino acid sequence information and position information to make representations for each protein. In prediction phase, we use deep neural network (DNN) modules composed of fully connected neural network layers to extract high-level feature information and make predictions. By designing this architecture, we can generate stronger vectorized representation for proteins and make more accurate predictions.

Our main contributions can be summarized as follows: (1) We use GCNs to capture the position information of the proteins in the PPIs networks graph, which can reflect the properties of proteins to some extent. (2) We propose a novel representation method that combines amino acid sequence information and position information. (3) We test our method on several benchmark datasets and the experimental results demonstrate the validity of our method. To the best of our knowledge, this is the first study that combines amino acid sequence information and position information to make representations for proteins.

## Experiments and results

In this section, we did two experiments to verify our model. In the first experiment, we compare our method with two state-of-the-art sequence-based PPIs prediction methods, including DPPI [5] and DeepFE-PPI [11]. DPPI takes a probabilistic sequence profile generated by PASI-BLAST as inputs and in prediction phase it uses 5 convolutional modules, 1 random projection module and 1 prediction module to extract features and make predictions. DeepFE-PPI adopts Word2vec to learn feature representation from a large protein database and in prediction phase it uses 4 fully connected layers to extract high-level feature. Through this experiment, it is confirmed that our method can achieve higher prediction accuracy than state-of-the-art methods.

In the second experiment, we did an ablation experiment by only using amino acid sequence information or position information to make predictions. The results of this experiment illustrate the effectiveness of our representation method, that is, combining amino acid sequence information and position information can generate a stronger representation for the protein.

### Dataset description

We test our method on three different benchmark datasets, including *Human* dataset, *Yeast* dataset and *S. cerevisiae* core dataset. *Human* and *Yeast* datasets are described by Profppikernel [14], which only contains the top-scoring physical interactions. To get the fair comparition results, we follow the same strategy as DPPI and remove redundancy of the *Human* and *Yeast* datasets such that no two PPIs are similar at sequence level. Two PPIs are considered similar if at least two sequences, each of one PPIs, have a sequence identity greater than 40%. *S. cerevisiae* core dataset is described by You et al. [2], which has a total of 11188 interactions, including 5594 positive interactions and 5594 negative interactions. The protein pairs which contain a protein with fewer than 50 residues or greater than 40% sequence identity are removed form the dataset. The amino acid sequences are retrieved from the Uniprot database (http://www.uniprot.org/).

**Fig. 1:**
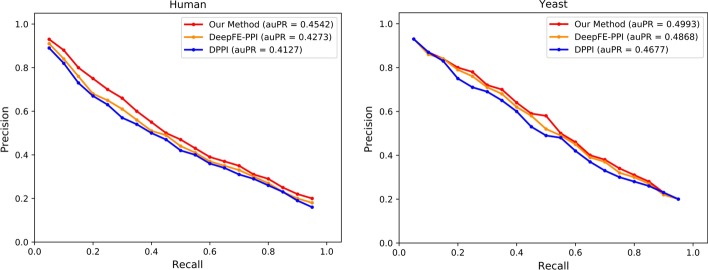
The performance comparison of our method with DPPI and DeepFE-PPI on *Human* and *Yeast* dataset. The auPR is the mean of 10-fold cross validation

**Table 1:** The performance comparison of our method with DPPI and DeepFE-PPI on *S. cerevisiae* core dataset

	Precision	Recall	Accuracy
DPPI	0.9668	0.9224	0.9455
DeepFE-PPI	0.9645	0.9299	0.9478
Our method	0.9702	0.9355	0.9533

The results are obtained by 5-fold cross validation

### Evaluation criteria

There are three common evaluation indicators in the classification problem, including accuracy, precision and recall. These indexes are defined as follows:

1 $$\begin{aligned} Accuracy= & {} \frac{T P+T N}{T P+T N+F P+F N} \end{aligned}$$

2 $$\begin{aligned} Precision= & {} \frac{T P}{T P+F P} \end{aligned}$$

3 $$\begin{aligned} Recall= & {} \frac{T P}{T P+F N} \end{aligned}$$

TP (true positive) is the number of samples that labels and predictions are both positive; TN (true negative) is the number of samples that labels and predictions are both negative; FP (false positive) is the number of samples that labels are negative but predictions are positive; FN (false negative) is the number of samples that labels are positive but predictions are negative.

And when label classes are not balanced, precision–recall curves are mainly used. The precision–recall curve shows the tradeoff between precision and recall. A large area under the curve represents both high recall and precision, the best case scenario for a classifier, showing a model that returns accurate results for the majority of classes it selects. Precision–recall curves give a more informative picture of an algorithm’s performance [15].

So when evaluating the method’s performance on *Human* and *Yeast* dataset, in which the number of negative samples is greater than positive samples, we follow the same strategy as DPPI, plot the precision–recall curves and compare the area under the curves of different methods. For *S. cerevisiae* core dataset, the number of negative samples is identical to that of positive samples, we compare accuracy, precision and recall of different methods.

### Parameter settings

When implementing our method, the model hyperparameters are determined by grid search and we get the best results with the following hyperparameters. The maximum length of protein amino acid sequence is set to 850 and when capturing proteins’ position information, we use one-layer GCNs to aggregate information from neighbor nodes. The number of neurons in the fully connected layers are 256, 128, 64, 32, 8 and 2 respectively. The dropout layer randomly drops neurons with a probability of 0.5. All parameters are updated by conducting stochastic gradient descent (SGD) and the learning rate of SGD is set to 0.01.

**Fig. 2:**
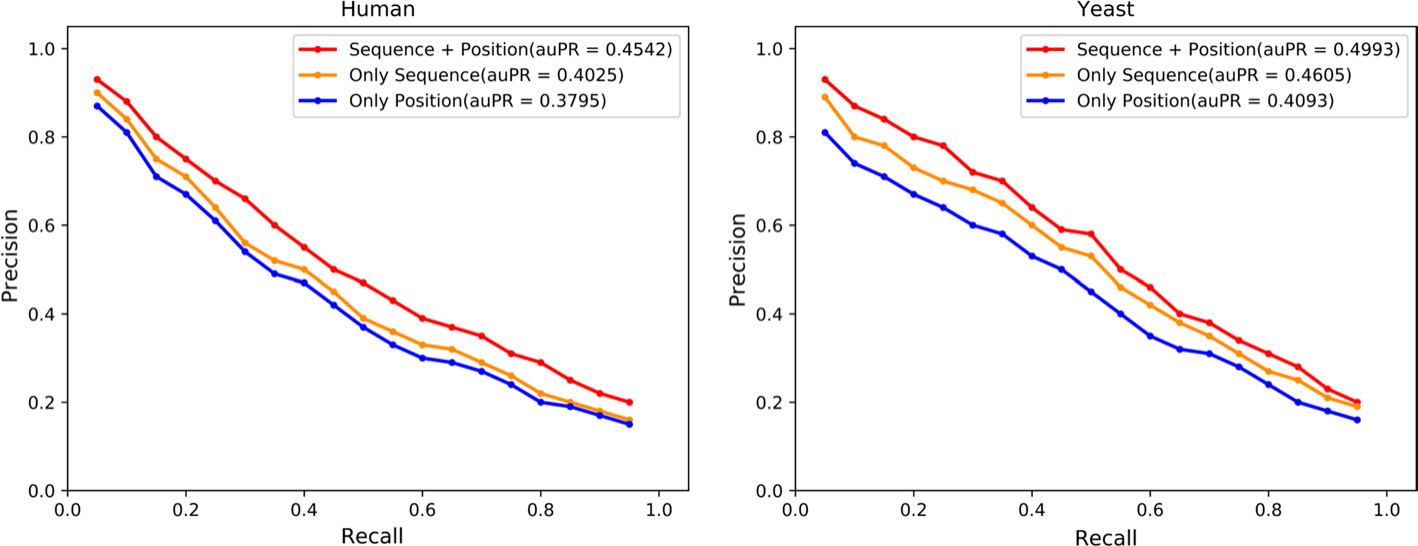
The ablation experiment on *Human* and *Yeast* datasets. We compare the performance of only using protein amino acid sequence information or position information to make predictions. The auPR is the mean of 10-fold cross validation

**Table 2:** The ablation experiment on *S. cerevisiae* core dataset

	Precision	Recall	Accuracy
Only Position	0.9004	0.9325	0.8992
Only Sequence	0.9587	0.9231	0.9372
Sequence + Position	0.9702	0.9355	0.9533

The results are obtained by 5-fold cross validation

### Results

First, we compare our method with two state-of-the-art sequence-based methods, DPPI and DeepFE-PPI. We perform 10-fold cross validation on *Human* and *Yeast* datasets and compute the mean auPR (area under precision–recall curve), the results are shown in Fig. 1. In addition, following the same strategy as DPPI and DeepFE-PPI, we perform 5-fold cross validation on *S. cerevisiae* core dataset and report their average results in terms of precision, recall and accuracy, the results are shown in Table 1.

Next we conduct an ablation experiment to confirm the effectiveness of combining two types of information, we make predictions by only using protein amino acid sequence information or position information. Same as the first experiment, we perform 10-fold cross validation on *Human* and *Yeast* datasets and compute the mean auPR, the results are shown in Fig. 2. On *S. cerevisiae* core dataset, we perform 5-fold cross validation and count the precision, recall and accuracy, results are shown in Table 2.

## Discussion

The results of the first experiment show that our method has an improvement over the previous methods. From Fig. 1, we can see that our method gets the best results on *Human* and *Yeast* datasets. On *Human* dataset, it achieves the largest mean auPR with the value of 0.4542 while DPPI is 0.4127 and DeepFE-PPI is 0.4273. Our method has 10.06% and 6.30% improvement over DPPI and DeepFE-PPI respectively. On *Yeast* dataset, our method also gets the largest mean auPR with the value of 0.4993 compared with DPPI’s 0.4677 and DeepFE-PPI’s 0.4868. It has 6.76% and 2.57% improvement over DPPI and DeepFE-PPI respectively. In addition, from Table 1, we can see that our method also achieves the best performance with 97.02% average precision, 93.55% average recall and 95.33% average accuracy on *S. cerevisiae* core dataset.

The first experiment proves the effectiveness of our method. From the above results, we can draw the conclusion that our method can make more accurate predictions. And compared with previous works, our method is simpler in representation phase. We use one-hot encoding to encode sequence information of protein and GCNs to capture position information, then we get the final representation matrix by combining them. While DPPI needs to generate a probabilistic sequence profile for each protein using PASI-BLAST and DeepFE-PPI needs to train a Word2vec model firstly and then uses the pretrained model to generate representation vector for each protein, which is complicated and time-consuming.

In the second experiment, we conduct an ablation experiment to compare different representation methods. We only use amino acid sequence information or position information to make predictions, and compare the prediction results with the method of combining these two types of information. Figure 2 shows that combining sequence information and position information achieves the best prediction results on *Human* dataset and *Yeast* dataset. The same conclusion holds on the *S. cerevisiae* core dataset, as can be seen from Table 2.

In addition, we can also observe that the prediction results obtained using only amino acid sequence information are more accurate than the prediction results obtained using only position information, indicating that amino acid sequence information is more important when representing proteins. Combining these two types of information gets the best prediction results, it confirms the correctness of our method that position information of protein in the PPIs networks graph can reflect the properties of proteins to some extent and is an important supplement to protein amino acid sequence information.

## Conclusion and future work

In this paper, we propose a novel method that combining sequence information and position information to generate representations for proteins. When capturing position information of proteins in the PPIs networks graph, we use GCNs to aggregate feature information of neighbor nodes. In prediction phase, we design DNN modules to extract high-level features and make predictions. We conduct extensive experiments on three different benchmark datasets to verify the effectiveness of our method, and carry out in-depth analysis of the experiment results. Continuing work will improve the design of DNN architecture to get a better prediction performance.

## Methods

PPIs prediction is essentially a classification problem and we need to identify that the given two proteins whether to interact. We train our model in a supervised way, it takes the representations of protein pairs as inputs and outputs a score representing the interaction probability. In order to illustrate our model clearly, we first introduce the principles and applications of GCNs. The following subsections will give further illustrations on the overall framework of our model, the method of encoding protein amino acid sequence information, the method of capturing proteins’ position informatin in the PPIs networks graph and the design of DNN modules in the prediction phase.

### Graph convolutional networks

Deep learning is a major advancement in the field of machine learning in recent years, which has aroused great interest of researchers and is widely used in several machine learning tasks, including computer vision, image analysis, speech recognition, information retrieval, natural language processing, reinforcement learning and multi-agent systems [16–23]. In addition, in the field of bioinformatics, deep learning is also widely used. For example, deep learning algorithms have been successfully applied to predict the association between *Human* diseases and microRNA, a type of non-coding RNA [24]. Compared with traditional machine learning methods, deep learning is suitable for processing and analyzing complex data, extracting and abstracting high-dimensional feature, which is helpful to process increasing amounts and dimensions of data generated by high throughput technique in bioinformatics.

**Fig. 3:**
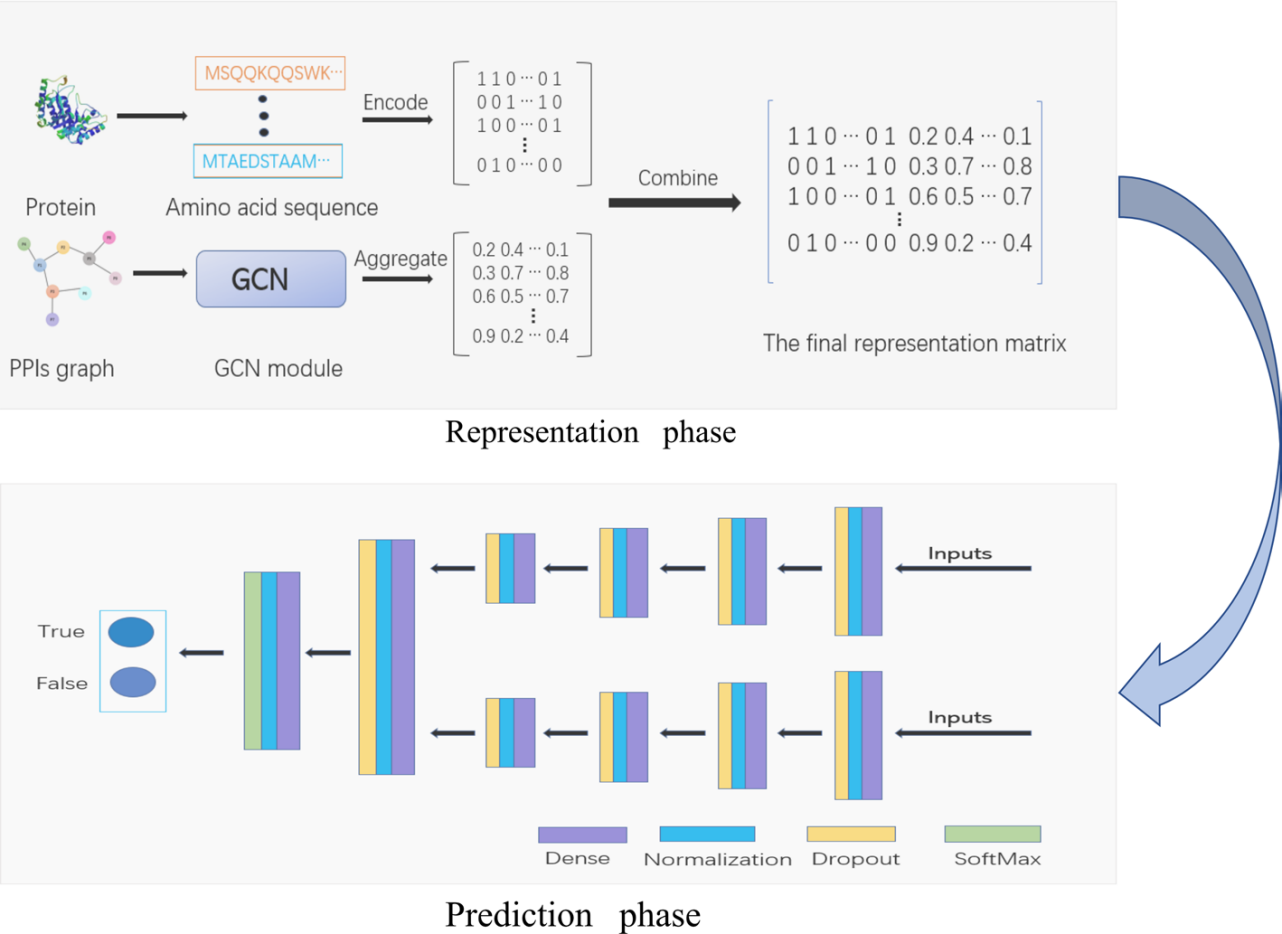
The framework of our method. There are two phases, representation phase and prediction phase. In representation phase, we apply GCNs to capture the position information and get the final representation matrix by combining sequence information and position information. In prediction phase, we take the representation matrix as inputs and use DNN modules to extract high-level features and make predictions

However, in the real world, lots of data is generated from non-Euclidean domains and represented as graphs with complex relationships and interdependency between nodes. If we can make full use of the information of the graph structure, it will be of great help to solve the problem. Such as modeling the multiagent coordination problems in cooperative environments under the networked social learning framework under four representative topologies [25].

The characteristics of data with graph structure can be summarized as follows. Each node in the graph can be regarded as an object with its own unique attributes. And nodes are connected by edges, indicating that there is a certain relationship between them. We need to comprehensively consider the attribute information of the node itself and the attribute information of its neighboring nodes to accurately make representations for nodes in graph-structured data.

In graph-structured data, the previous deep learning algorithm can not be directly applied. To solve the problem, researchers generalize the operation of convolution from traditional data to graph-structured data and propose GCNs, which generate the node’s representation by aggregating feature information of its neighbors [26]. GCNs layer aggregates information from connected nodes to generate hidden representation for center nodes and then a non-linear transformation is applied to the hidden representation. By stacking multiple GCNs layers, the final hidden representation of each node receives message from further neighbors. The rule of aggregating neighbors’ feature information in vector form can be described by Eq. 4,

4 $$\begin{aligned} h_{i}^{(l+1)}=\sigma \left( \sum _{j \in N_{i}} \frac{1}{c_{i j}} h_{j}^{(l)} W^{(l)}\right) \end{aligned}$$

where $h_{i}^{(l+1)}$ is the hidden representation of node *i* in the $(l+1)th$ layer, $N_{i}$ is the set of node $i's$ neighbors, $C_{i j}$ is an appropriately chosen normalization constant for the edge $\left( v_{i}, v_{j}\right)$, $W^{(l)}$ is a layer-specific weight matrix and $\sigma (\cdot )$ denotes a non-linear activation function.

GCNs take the graph structure information and node feature information as inputs and the outputs of GCNs can be different mechanisms according to different graph analytics task, including node-level, edge-level and graph-level [26].

GCNs are widely used in the processing of graph structure data, and have achieved excellent performance compared with previous methods. For example, in a citation network, partial nodes are labeled and others are unlabeled, using GCNs can learn an appropriate representation for each node, which is very important for predicting labels of the unlabeled nodes [27]. Similarly, there is also work proposing an improved spectral-based GCNs, which can work directly on directed graph data in semi-supervised nodes classification tasks [28]. In addition, GCNs are used to make multi-relational link prediction in a multimodal graph [29]. There is also work applying GCNs in the protein interface prediction problem, but different from ours, they represent a protein as a graph where each amino acid residue is a node whose features represent the properties of the residue [30]. In our paper, we represent the PPIs networks as a graph where each protein is a node. We use GCNs to capture position information of protein in the graph, which can be an important supplement to protein amino acid sequence information.

### Design of the proposed model

In Fig. 3, we demonstrate the flow diagram of our method. The integrated flow diagram has two phases, representation phase and prediction phase. In representation phase, we process proteins’ amino acid sequence information and PPIs networks graph information to generate representation for each protein. In prediction phase, we take the final representation matrix as inputs, which combines amino acid sequence information and position information, and use DNN modules to extract high-level features and make predictions. Next, we will give details of the methods used in the representation and prediction phases.

### One-hot encoding

One-hot encoding, also known as one-bit effective encoding, mainly uses bit status registers to encode each state. Each state is controlled by its own independent register bit, and only one bit is valid at any time. In practical machine learning applications, features are not always continuous values, and may be some categorical values. For such features, we usually need to digitize the features. For example, gender is a categorical attribute with two possible values, male or female. We can use [1, 0] for male and [0, 1] for female using one-hot encoding. One-hot encoding solves the problem that the classifier is not good at handling attribute data, and it also plays a role of expanding features to a certain extent.

### Encode amino acid sequence information

Proteins are chains of amino acids that fold into a three dimensional structure that gives them their biochemical function. There are 20 different types of amino acids in organism. In this paper, we use one-hot encoding to encode amino acids, so each amino acid can be represented by a 20-dimensional vector consisting of 0 and 1.

The detailed process is as follows, we use natural numbers ranging from 1 to 20 to give each unique amino acid an identity, and convert the original amino acid sequence to a vector of natural numbers. We construct an identity matrix with shape of $20*20$, every line in the matrix is the feature vector of a unique amino acid. Then we can convert the identity vector to feature vector by looking up in the identity matrix. After that, we get the amino acid sequence information of protein in the vector form, and for the convenience of subsequent processing, we set a fixed length for the obtained vector. If its length is less than the fixed length, we pad zeros to the front of the sequence and if its length is longer than the fixed length, we truncate the sequence in the front. In the previous methods, when encoding protein sequence information, the representation vector of the protein is usually a trainable parameter. Our method is more simple and efficient, and there are no parameters that need to be trained.

### Capture proteins’ position information

**Fig. 4:**
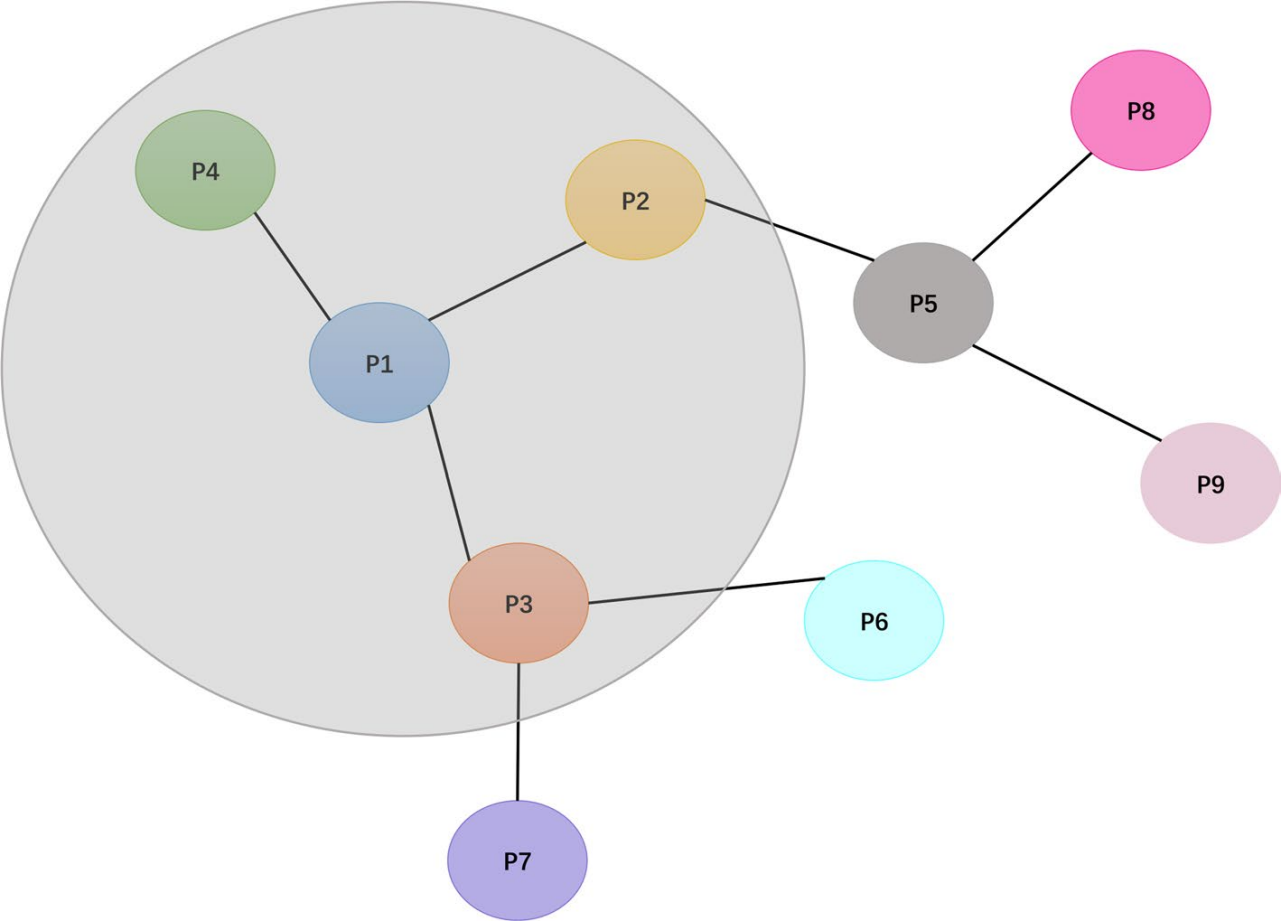
The PPIs networks graph. The node in the graph represents protein, and the edge between two nodes represents the protein–protein interaction (for example, protein ‘P1’ interacts with ‘P2’, ‘P3’ and ‘P4’)

In our method, we model the PPIs networks as an unweighted and undirected graph where each protein is a node, as shown in Fig. 4. The edge between two nodes indicates that these two proteins can interact. The position information of protein in the PPIs networks graph reflects which proteins it can interact with, which is essentially a reflection of the protein’s characteristics. Take protein ‘P1’ as an example, it interacts with ‘P2’, ‘P3’ and ‘P4’. So when capturing position information of protein ’P1’, we use GCNs to aggregate information about its neighbor nodes ’P2’, ’P3’ and ’P4’.

In our model, we take the graph structure information and node feature information as inputs. The graph structure information mainly includes the adjacency matrix and degree matrix of the graph, where the adjacency matrix describes the relationship between the nodes in the graph, and the degree matrix describes the number of connecting nodes of each node. The dimensions of these two matrices are determined by the number of nodes in the graph. Adjacency matrix and degree matrix are constructed based on the training set, without using data of testing set, so there isn’t a label leakage problem. The node feature information is also a matrix, where each row represents the feature information of a node, the number of rows is determined by the number of nodes, and the number of columns is the dimension of the feature vector of each node. Here we use one-hot encoding to encode each unique protein, so the dimension of the feature vector of each protein is the same as the number of proteins.

The way of GCNs capturing position information for each protein in the graph can be expressed by Eq. 5.

5 $$\begin{aligned} X_{1}^{N * f}=\sigma \left( \widetilde{D}^{-1} \tilde{A} X_{0}^{N * N} W_{0}\right) \end{aligned}$$

In our work, one-layer GCNs achieve the best performance. Here, $\tilde{A}=A+I_{N}$ is the adjacency matrix of the PPIs networks graph with added self-connections, $I_{N}$ is the identity matrix. $\widetilde{D}_{i i}=\sum _{j} \tilde{A}_{i j}$ is the degree matrix, $W_{0}$ is the trainable weight matrix in the first layer and $\sigma (\cdot )$ denotes an activation function, such as $ReLU (\cdot )$. $X_{0}^{N*N}$ is the original feature matrix, *N* is the number of proteins in the graph, here we use one-hot encoding to encode each unique protein, so the original feature matrix is the identity matrix with shape of $N*N$. $X_{1}^{N*f}$ is the output feature matrix, *f* is the feature length of each node after GCNs operation. So after the GCNs operation, each node’s hidden representation is composed of its original feature information and first-order neighbor’s feature information, which contains the position information of the protein in the PPIs networks graph.

### Design of DNN modules

To combine amino acid sequence information and position information, we concatenate the above two matrices from the amino acid sequence information and position information and get the final representation matrix, where each row is a feature vector of an unique protein. We take the final representation matrix as inputs of DNN modules. There are two separate DNN modules, each module processes one protein of the input pairs. These two DNN modules have the same structure, consisting of 4 fully connected neural network layers, 4 normalization neural network layers and 4 dropout neural network layers to extract high-level features that hidden in embedding vectors.

As an important achievement of deep learning in recent years, batch normalization has been widely proven to be effective and important. During model training, batch normalization uses the mean and standard deviation on small batches to continuously adjust the intermediate output of the neural network, so that the value of the intermediate output of the entire neural network in each layer is more stable. The use of batch normalization can make the convergence faster, the total training time is shorter and the effect is improved.

Dropout is another important trick widely used in deep learning, it means that during the training process of deep neural networks, the neural network unit is temporarily dropped from the network with a certain probability. For machine learning models, if the model has too many parameters and too few training samples, the trained model is prone to be overfitting. Dropout can effectively alleviate the problem of overfitting and improve the generalization ability of the model.

After extracting the features of the two proteins, we concatenate last hidden vectors of the both DNN modules. Then the concatenated vector is processed by a joint module composed of 2 fully connected neural network layers, and a softmax layer is used to predict interaction probability.

## Data Availability

The datasets analysed during the current study are available in the zenodo repository, https://doi.org/10.5281/zenodo.3960077.

## Abbreviations

PPIs: Protein–protein interactions
GCNs: Graph convolutional networks
DNN: Deep neural network
SGD: Stochastic gradient descent
auPR: Area under precision–recall curve
